# ASO Author Reflections: Bridging the Gap: Patient-Centered Multi-level Solutions to Address Disparities in Foregut Cancer Care

**DOI:** 10.1245/s10434-025-17262-4

**Published:** 2025-03-29

**Authors:** Ioannis Liapis, Martin J. Heslin, Annabelle L. Fonseca

**Affiliations:** Department of Surgery, University of Alabama at Birmingham, Birmingham, AL USA

## Past

Disparities in foregut cancer care remain a significant challenge, particularly in the southeastern United States, where socioeconomic disadvantage, limited availability of specialty care, and systemic barriers intersect to limit access to high-quality cancer care. While prior quantitative studies have highlighted the significant role of race, socioeconomic status, and healthcare access in shaping these disparities,^1–3^ there has been a gap in understanding this from the patient’s perspective—the barriers they experience, and how these barriers shape their engagement with the healthcare system.

Gaining insight into the patient perspectives and the lived experiences of patients navigating foregut cancer care is key to a more comprehensive understanding of the barriers that exist, and modifiable factors that may be targeted for interventions. This study sought to address the existing knowledge gap by integrating qualitative insights with quantitative data to explore barriers to foregut cancer care from the patient perspective.^4^

## Present

This mixed-methods study integrated patient narratives with data from the electronic health record to gain a more nuanced understanding of the challenges patients face in accessing and adherent to treatment for foregut cancer. Patients described significant challenges related to financial burden, transportation, lack of primary care engagement, and inadequate social support, all of which contributed to delays in care. A striking finding was that many patients did not recognize these delays as abnormal, illustrating how suboptimal access to care may have become normalized in historically underserved communities. In addition, physician-patient communication emerged as a recurrent theme, with complex medical terminology and inconsistent messaging across different providers resulting in eroded trust and decreased treatment adherence.

In addition to individual and interpersonal level barriers, this study also highlighted the role of healthcare system limitations including inadequate health system infrastructure, fragmented referral pathways and the logistical challenges associated with receiving cancer care at a safety-net hospital and tertiary referral center. From a systemic perspective, the high proportion of uninsured patients in this study and the barriers they face emphasizes the many structural challenges facing disadvantaged populations. Previous research has shown that Medicaid expansion could mitigate some of these disparities by increasing access to cancer care and reducing the financial burden to both patients and healthcare institutions.^5^ Unfortunately, the compounding effects of socioeconomic deprivation, limited insurance coverage, rural residence, and lack of specialty care creates and perpetuates a cycle of delayed care, mistrust, and suboptimal treatment adherence, ultimately leading to worse oncologic outcomes. These barriers not only hinder timely diagnosis and initiation of treatment but also erode patient confidence in the healthcare system, reinforcing disparities in survival and quality of life for individuals with foregut cancers. However, many of these barriers are modifiable. Targeted interventions, such as patient-centered communication strategies, patient navigation programs, health literacy initiatives, strengthening community engagement, and policy efforts to expand insurance coverage, can help bridge these gaps.

## Future

While this study offers valuable insights into the barriers faced by patients with foregut cancer, addressing the barriers it highlights will require targeted interventions at multiple levels. At the institutional level, the implementation of strategies such as expanding telehealth services, patient navigation programs, and strengthening partnerships with the community may help reduce delays in referrals. In addition to improving provider communication, simplifying patient-facing educational materials and the use of health literacy appropriate materials can help bridge communication gaps. Collaborating with community leaders and expanding the use of patient navigators, particularly those from similar communities, could be critical to increasing patient trust. From a policy perspective, broader adoption of Medicaid could significantly improve access to cancer care for a large number of patients, by improving patient access, as well as by providing an avenue for safety-net institutions to fund patient-centered services.

Future research should focus on longitudinal assessments of how barriers evolve over time and the effectiveness of targeted interventions in improving access to treatment. Expanding the scope of research to include the perspectives of caregivers and healthcare providers could provide a more comprehensive understanding of the multi-level barriers to high-quality cancer care. Caregivers play a vital role in treatment adherence and navigating the healthcare system, yet their perspectives remain underrepresented in the literature. Likewise, gaining insights from oncologists, community physicians, primary care physicians and healthcare administrators could help identify institutional constraints and opportunities. Evaluating the impact of proposed potential health system interventions on patient reported outcomes and survival will provide the data needed to guide future policy efforts.

In conclusion, our study highlights the multifaceted nature of barriers that patients with foregut cancer encounter in accessing and adhering to treatment. Addressing these barriers requires comprehensive, multilevel interventions that integrate patient-centered approaches, institutional initiatives to improve care delivery, and broader policy efforts. By aligning these efforts, we can expand access to high-quality, equitable cancer care for many patients.
